# Advances in Structural and Morphological Characterization of Thin Magnetic Films: A Review

**DOI:** 10.3390/ma16237331

**Published:** 2023-11-24

**Authors:** Payel Aich, Carlo Meneghini, Luca Tortora

**Affiliations:** 1 Department of Science, Roma Tre University, Via della Vasca Navale 84, 00146 Rome, Italy; payel.aich@uniroma3.it (P.A.); luca.tortora@uniroma3.it (L.T.); 2 LASR3 Surface Analysis Laboratory Roma Tre, Via della Vasca Navale 84, 00146 Rome, Italy

**Keywords:** microscopy, diffraction, spectroscopy, thin magnetic films, atomic structure

## Abstract

The present review places emphasis on a comprehensive survey of experimental techniques to probe the structural and morphological features at the nanoscale range in thin magnetic films, incorporating those available at in-house laboratories as well as those at state-of-the-art synchrotron radiation facilities. This elucidating the range of available techniques, and the information they can yield represents a step for advancing the understanding of and for unlocking new possibilities in the design and optimization of thin magnetic films across a wide range of applications.

## 1. Introduction

Magnetic thin and ultrathin films are materials with a thickness of the order of a few nanometers or less, down to the atomic monolayers, exhibiting unique magnetic properties with respect to their bulk form mainly originating from their reduced dimensionality and interface phenomena [1,2,3]. The low dimensionality along with intrinsic anisotropy promotes the occurrence of phenomena that are fascinating for the fundamental physics of magnetic systems and important for technologically advanced applications, ranging from magnetic storage devices to sensors. The competition between morphological, structural and compositional degrees of freedom makes reliably understanding the origin of the macroscopic magnetic properties of these systems a challenging task that requires a systemic approach, combining state-of-the-art and complementary investigative techniques, which are the subject of this review. Today, the range of magnetic films is incredibly wide, from single layer to multilayer, thin to ultrathin, and buried layer to surface films. This has forced us to limit the scope of this review to the major techniques for the morphological, atomic and electronic characterization of thin and ultrathin magnetic single layers.

The initial drive behind the exploration of magnetic thin films was their potential to enhance data storage capacity, particularly for high and ultra-high densities. By manipulating the magnetization of these films, information can be encoded and retrieved in the form of magnetic bits. In this context, perpendicular magnetic anisotropy (PMA) has been identified for offering the smallest bit size and the highest storage capacity. Consequently, magnetic thin films have been developed as the active layer for data storage in magnetic recording media and magnetic memories [4]. In recent years, there has been a growing interest in investigating novel materials and fabrication techniques aimed at the advancement of magnetic thin-film technology. By exploring new materials, morphologies and interfacial interactions, new properties and unique behavior have emerged, opening up new technological possibilities. Magnetic thin films have become indispensable components in spintronic, magnetoelectronic and magneto-optic devices. They are utilized in spin valves, magnetic tunnel junctions and spintronic sensors, where their unique magnetic properties are essential for manipulating electron spins and detecting magnetic fields. To achieve improved magnetic properties, such as higher magnetic anisotropy, reduced magnetization noise and enhanced thermal stability, various materials, like magnetic alloys, multilayers and nanostructured films are being investigated. Table 1 displays a list of classified magnetic thin films.

As film thicknesses are reduced to nano and sub-nano scale dimensions, interfacial effects profoundly influence the intrinsic magnetic properties due to broken symmetry and altered electronic structure at boundaries [44,45,46,47,48,49,50,51,52]. Key interfacial phenomena include lattice strain accommodation via misfit dislocations, interdiffusion-driven atomic rearrangements, spin-dependent electron scattering, and exchange coupling across heterojunctions. In fact, buried layers in the magnetic thin films can alter magnetization reversal mechanisms by breaking exchange coupling, modify magnetic anisotropy by strain-induced symmetry breaking and even provide electrical insulation between magnetic layers in tunnel junctions. Also, the final stoichiometric ratio of the magnetic compound manipulates the resultant properties exhibited by the thin films [53,54]. Hence, elucidating the structure-property relationships necessitates the precise control and advanced characterization of interfaces at the atomic scale.

Various physical vapor deposition techniques are employed for thin-film growth, including molecular beam epitaxy (MBE), chemical vapor deposition (CVD), pulsed laser deposition (PLD), magnetron sputtering, electrodeposition (electroplating), atomic layer deposition, spin coating and sol gel synthesis [55,56,57,58]. Different growth methods enable different ways to tune thickness, crystallinity, texture and stoichiometry, and defect densities by controlling kinetic and thermodynamic parameters during synthesis. Chemical methods generally allow for mass production, while physical methods, such as epitaxial growth, are more research-oriented, allowing for the precise engineering of abrupt interfaces and magnetic exchange coupling by matching lattice constants between magnetic layers and substrate and capping layers.

Considering all the above, quantitative morphological, structural and coordination chemistry analysis is thus imperative to reliably relate magnetic properties with the underlying structural landscape. The scope of this review is based on the state-of-the-art techniques available to date for the study of magnetic thin films, both in the laboratory (abbreviated as Lab. in the text below) and at large research facilities, such as synchrotron light sources (abbreviated as Sync. in the text below). Characterization techniques can be grouped into three main classes according to the information that can be obtained: morphological characterization techniques, scattering-based techniques, and spectroscopic-based techniques. Morphological information is generally derived from imaging tools such as microscopy-based techniques. Scattering techniques involving diffraction, small-angle scattering and reflectivity are generally based on X-ray or neutron radiations, they provide information about crystallographic phases, layer thickness, interface roughness and atomic structure in the film. Spectroscopic studies look at the response of the system as a function of energy and, depending on the probe used—electron, photon, neutron—provide chemically selective details of local composition, coordination chemistry, electronic structure and magnetization state. We will discuss the main techniques with specific examples below. This will enable the reader to navigate through a wide range of possibilities and identify specific combinations of techniques that are appropriate for their interests.

## 2. Imaging Techniques for Morphological Characterization

Microscopy imaging techniques are critical for the morphological characterization of thin films because they provide nanoscale structural and compositional information that impacts properties. Techniques discussed below reveal grain size, grain boundaries, surface roughness and interfaces, and defects at high resolution. When coupled with spectroscopy, microscopy may provide compositional mapping and magnetic information. This multiscale morphological information is key to elucidating structure–property relationships in thin films.

### 2.1. Scanning Transmission Electron Microscopy (TEM)

**Figure 1:**
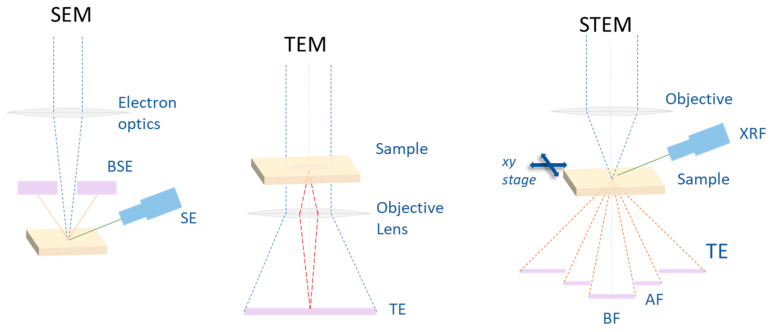
Main features of different electron microscopy setups. In SEM, the electron beam is focused on the sample, the image is reconstructed from secondary (SEs) and backscattered electrons (BSEs). In TEM, the sample is illuminated by a parallel electron beam, and the 2D image (transmitted electron (TE)) is formed on the detector. In STEM, the electron beam is focused on the sample, and transmitted electrons are visualized on the detectors. BF: bright field; AF: annular dark field. The X-ray fluorescence (XRF) allows for chemical maps.

Transmission electron microscopy (TEM) is a powerful materials characterization technique that utilizes a high-energy electron beam transmitted through a thin specimen (see Figure 1). The interactions between the incident electrons and the sample provide information about the microstructure, crystal structure, composition and electronic properties with extremely high spatial resolution. In the TEM electromagnetic optics, the high energy (100–300 keV) electrons are generated from an electron gun in the form of a thin, coherent beam that is then transmitted through tin samples. The electrons interact with the atoms of the specimen in different ways; they can be elastically scattered, partially absorbed or diffracted by crystalline planes. The transmitted electrons can be analyzed by various detectors to provide accurate information about atomic arrangement, crystalline phases and defects, allowing for the achievement of structural, chemical and electronic characterization across length scales ranging from the atomic scale crystallography to the microstructure. Advanced TEMs are capable of an atomic-scale resolution below 0.5 Å, which is especially suited to probe thin film interfaces, defects, grain structure, orientations, dislocations, and more [59,60]. However, due to the relatively short mean free path of electrons in matter, samples must be suitably prepared as thin slices, so to study thin films, it is necessary to cut thin cross-sections of the samples (cross-sectional TEM (XTEM)) using, for example, a focused ion beam (FIB) facility.

In addition to conventional TEM, there are several specific applications of TEM-based techniques that can be suitably exploited for studying thin magnetic films and their interfaces. Scanning TEM (STEM) is like conventional TEM, but it scans a focused electron beam to reconstruct images. STEM can utilize different modes like bright field, dark field and high-angle annular dark field (HAADF) imaging to provide contrast based on composition, thickness, diffraction, etc. [61,62]. Energy dispersive spectroscopy (EDS) allows for chemical analysis in TEM by measuring X-rays fluorescence photons emitted when the electron beam excites atoms in the sample. This information is crucial for understanding the elemental composition, segregation, composition gradients and the presence of impurities within the film. Electron energy loss spectroscopy (EELS) analyzes energy losses of the transmitted electron beam due to inelastic scattering, thus providing chemical bonding, valence, and core-level information. It can be used to study the chemical composition, oxidation state and electronic properties of different elements in thin films. TEM electron diffraction (ED) patterns give insights into crystal structure, orientation, epitaxial relations and phase at interfaces. Convergent-beam electron diffraction (CBED) allows for the probing of the local crystallographic structure at a nanoscale resolution. High-resolution TEM (HRTEM) can directly image atomic structures, lattice spacings and defects, and can be specifically useful to resolve atomic structure at interfaces. Lorentz TEM is used to image magnetic domain structures.

### 2.2. Scanning Electron Microscopy (SEM)

It is an essential characterization tool for understanding the structural and morphological properties of magnetic thin films. SEM utilizes a focused beam of electrons to image the sample surface (see Figure 1), providing nanometer-scale resolution. When coupled with elemental analysis techniques like energy-dispersive spectroscopy (EDS), SEM enables the determination of correlating composition and morphology.

For magnetic thin films, key aspects studied via SEM include grain size, grain boundaries, film continuity and surface roughness. Grain size impacts magnetic properties like coercivity and anisotropy. Smaller grains lead to more grain boundaries, which act as pinning sites for domain wall motion, increasing coercivity. Anisotropy depends on crystal symmetry, so larger grains with fewer defects promote magnetocrystalline anisotropy. Film continuity is critical for properties like giant magnetoresistance in multilayers. Surface roughness affects domain structure and impacts magnetic behavior. SEM can also directly image magnetic domain patterns in the film when combined with electron backscatter diffraction (EBSD). The recent reviews on SEM analysis have been listed in Table 2.

### 2.3. Time-of-Flight Secondary Ion Mass Spectrometry (ToF-SIMS)

**Figure 2:**
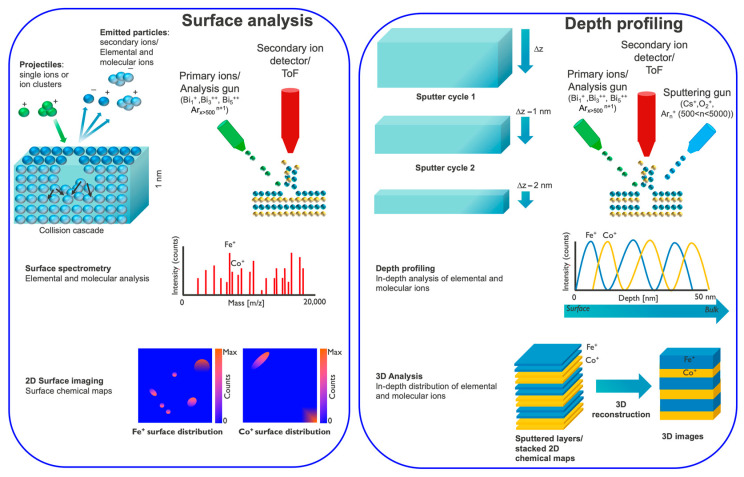
TOF-SIMS working principle and decoding data acquisition.

Mass spectrometry is an analytical technique that uses a mass spectrometer to separate ions by their mass-to-charge ratio. It is a powerful yet (micro-)destructive tool that provides information about a given sample from both its surface (with an overall sensitivity of parts per million (ppm)) and bulk (with a sensitivity of part per billion (ppb)) in comparison to EDS, AES (Auger electron spectroscopy) and XPS [130]. It provides a detailed analysis of a thin film’s chemical composition, as well as interfacial characterization, and yields information about the elemental distribution in the range of a few monolayers (1–3 monolayers) through depth profiling. Furthermore, by using advanced liquid-metal ion guns (LMIGs), one can reach a lateral resolution of about 100 nm for elemental detection (ToF-SIMS imaging). The average lateral resolution obtained is <50 nm with a depth resolution of <10 nm [67,69,131,132,133,134,135,136,137]. For thin films, static ToF-SIMS is helpful to detect any kind of inhomogeneity and defects on the surface as it provides chemical maps with high sensitivity, high mass resolution (M/ΔM 10,000) and a high lateral resolution. The depth profiling can provide information regarding anomalies in the thin-film layers and the interfaces. A three-dimensional image of the composition of film is also achieved, showing the possibilities of any diffusion across the interfaces (see Figure 2).

### 2.4. Spin-Polarized Low-Energy Electron Microscopy (SPLEEM)

For Low-Energy Electron Microscopy (LEEM), surfaces are imaged with the elastically backscattering of coherent low-energy electron beams that produce a high reflected electron intensity [70,138]. SPLEEM is a LEEM technique modified by the incorporation of spin-polarized GaAs-based electron gun. It is a unique and powerful tool to investigate the surface magnetic properties for low-dimensional magnetism along with in-situ measurements [70,72,139]. The basis of the magnetization sensitivity in SPLEEM is the exchange interaction between incident spin-polarized electrons and the target electrons in the magnetic sample, which provides a three-dimensional magnetic vector mapping of nanostructures. The spin-polarized incident electrons are reflected at normal incidence from the surface in a manner that depends upon the relative orientation of the spin polarization $\vec{P}$ of the electron beam and the local magnetization $\vec{M}$ in the surface layers of the sample. The reflected electrons, whose polarization is not measured, are used for imaging. The magnetic contrast (proportional to $\vec{P}\cdot \vec{M}$) is usually superimposed on the structural contrast and can be easily disentangled using the difference between the images taken with opposite $\vec{P}$ values. Cathode lenses have large chromatic and spherical aberrations that limit the lateral resolution to some 10^1^ nm. The depth resolution is, however, higher thanks to Fabry–Perot-type interferometers that allows for a thickness resolution in thin films down to the atomic scale. The main limitations of SPLEEM are that it requires crystalline materials with a strong preferred crystal orientation that can produce a specular beam along the optical axis of the microscope to achieve a suitable resolution.

SPLEEM has been used to address aspects of magnetic domain and domain wall structure in thin magnetic films and surface-supported nanostructures [140,141,142,143], exchange coupling in ferromagnetic/nonferromagnetic sandwiches [144,145] and spin reorientation transition in Co layers or Fe/Ni bilayers [146,147]. SPLEEM has also been used to study spin-dependent electron reflectivity and spin-resolved quantum well resonances in ferromagnetic Fe and Co magnetic thin films [148,149].

### 2.5. Photoemission Electron Microscopy (PEEM)

Photoemission Electron Microscopy (PEEM) is a powerful imaging technique. It combines photoelectron spectroscopies with microscopic techniques and is specifically useful to study thin magnetic films and interfaces [150,151,152,153,154]. PEEM was initially developed with laboratory sources (UV), but brilliant soft X-ray synchrotron sources allow for greater element specificity. The basic of PEEM technique involves exciting the sample with UV or soft X-ray and providing an image of the position from which the photoelectrons emerge from the sample. Owing to the relatively short photoemitted electron mean free path, PEEM is mainly a close-to-surface (2–5 nm depth) sensitive technique. The spatial resolution limits the aberrations in the electron optics as well as the electron mean free paths in the few ten nm range. Magnetic sensitivity is obtained using a polarized incoming beam, making PEEM sensitive to the spin and orbital occupancy of the electronic states close to the sample surface. In the soft X-ray energy range, being 200–2000 eV, absorption is dominated by electronic transitions to unfilled 3d (transition metals) or 4f (rare earths) bands where the magnetism originates, making the photoelectrons very sensitive to the magnetism of the sample. When circularly polarized radiation is used, the X-ray circular magnetic dichroism (XMCD) effect, originating from the unbalanced filling of spin-up and spin-down states, provides additional sensitivity to the magnetic state of the sample [155,156].

### 2.6. Atomic Force Microscopy (AFM)

**Figure 3:**
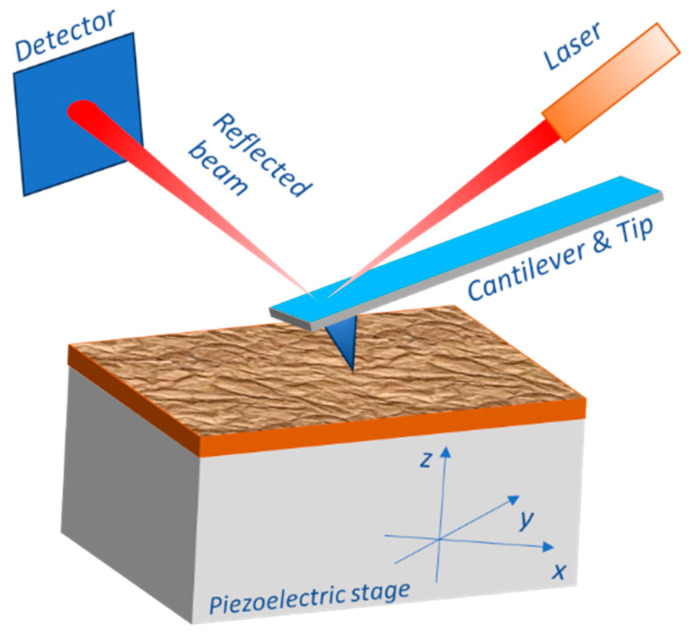
Working principle of AFM. It utilizes a tiny cantilever with a sharp tip at one end. The deflection of the cantilever is recorded looking at the reflection of the laser beam and is used to create a map of the surface morphology.

Atomic force microscopy (AFM) has become an indispensable tool for the characterization and development of thin-film materials and devices, allowing for the wide probing of several surfaces and interfaces properties with nanoscale resolution. In AFM, a sharp tip on the end of a flexible cantilever scans across the sample surface while deflections of the cantilever are measured (Figure 3). These deflections correspond to tip-sample interaction forces that allow for the generation of topological images of the sample surface. AFM is ideally suited for characterizing thin films and buried interfaces as it can probe nanoscale morphology, surface roughness, friction, magnetism, electrical properties, and more [157,158,159,160]. Operating modes such as contact, tapping, and non-contact AFM allow for the optimized imaging of delicate samples. Key strengths include the ability to map topological and material variations with nanometer resolution, measure extremely weak forces, and conduct the in situ imaging of samples during processing. Advanced AFM techniques can provide compositional mapping, phase contrast, electronic structure, and even sub-molecular resolution on suitable samples. Specific applications of AFM on thin magnetic films are detailed in Table 3.

The main use of AFM is for surface topography. It can provide high-resolution images of thin films, revealing their surface roughness, grain boundaries and other surface features. By analyzing the height profile obtained from AFM images, the film thickness can be determined with nanometer-level precision. This is particularly useful for monitoring the growth or deposition process of thin films. By analyzing the force field, AFM can map the nanomechanical properties of thin films, such as their elastic modulus, adhesion and hardness. By exploiting different modes of AFM, such as force spectroscopy or nanoindentation, the local mechanical response of the film can be measured. This information is crucial for understanding the film’s behavior under different conditions and for optimizing its performance.

Advanced AFM modes provide specific applications addressed to study magnetic nanostructures, giving a wealth of information critical for understanding the magnetic behavior of these thin films and interfaces. Magnetic force microscopy (MFM) is a specialized AFM mode that detects long-range magnetic forces between a magnetic tip and the sample surface. This method allows for the imaging of magnetic domain structures down to some 10^1^ nm scale resolution. Magnetic exchange force microscopy (MExFM) directly provides 2D maps describing the variation of magnetic exchange coupling strength. In this technique, a non-magnetic tip is used, and the interaction between the probe and the sample arises from the magnetic exchange coupling between the probe and the magnetic sample. The spin-polarized scanning tunneling microscopy (SP-STM) uses a magnetic tip to probe magnetic states with atomic resolution. It can directly observe nanoscale spin textures at surfaces and interfaces. Furthermore, 2D magnetic imaging can be performed while simultaneously applying electric or magnetic fields to reveal dynamic switching behavior. High-resolution AFM techniques can directly observe domain wall structure, pinning, and motion at surfaces and buried layers.

To conclude the section of imaging methods, Table 4 provides a survey of the different imaging techniques used till date to assess the magnetic domains of the thin films.

## 3. X-ray-Scattering Techniques for Thin Films and Interfaces

Scattering-based techniques rely on the interference of waves (radiation or particles) scattered from the sample. We focus here on X-ray-scattering techniques because neutron techniques require dedicated large-scale facilities, and electron diffraction is mainly focused on surface analysis due to the specific surface sensitivity of the electron probes. Although we notice that neutron-based techniques can be considered thanks to the specific sensitivity of neutrons to magnetic momentum, electron diffraction is routinely available at TEM facilities on suitably prepared thin-section samples.

**Figure 4:**
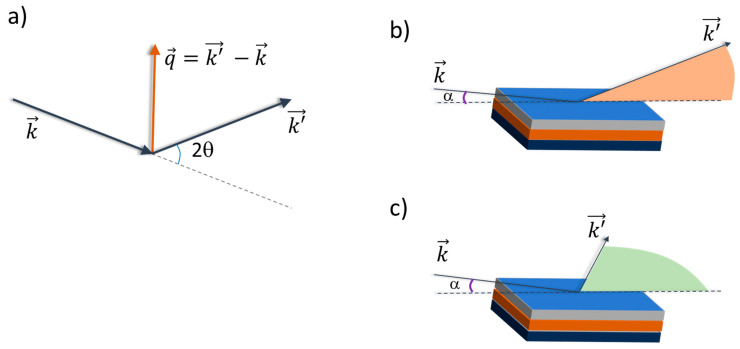
(**a**) Elastic scattering geometry and definition of the scattering (exchanged momentum) vector $\vec{q}$. (**b**,**c**) Grazing incidence X-ray diffraction (GIXRD) geometries enhance surface sensitivity. The out-of-plane diffraction geometry (**b**) has $\vec{q}$ roughly perpendicular to the film plane, therefore providing structural information perpendicular to the film plane. The in-plane diffraction geometry (**c**) has $\vec{q}$ roughly parallel to the film plane, therefore providing structural information parallel to the film plane.

The fundamental principle of X-ray scattering and diffraction (XRD) is deeply and accurately discussed elsewhere [172,173]. In periodic structures (crystals and multilayers), diffraction maxima are found for the following:

(1) $$\vec{q}\cdot \vec{d}=2n\pi$$

where $\vec{d}$ is the periodicity vector, $\vec{q}=\vec{k^{\prime }}-\vec{k}$ is the scattering vector, $\vec{k}$ and $\vec{k^{\prime }}$ are the incoming and diffracted beam wavevectors (Figure 4), and $|k|=|k^{\prime }|=\frac{2\pi }{\lambda }$ representing elastic scattering. Equation (1) shows that diffraction probes periodic structures that are parallel to the scattering vector. Because $\left|q\right|=\frac{4\pi \sin\theta }{\lambda }$, Equation (1) is equivalent to the well-known Bragg equation: 2dsin⁡θ=nλ. Wide-angle (or high *q*) methods are used to provide crystallographic information in terms of unit cell symmetry and structure (average atomic positions in the unit cell). XRD is a well-known, laboratory available, non-destructive technique used primarily for the phase identification of crystal structures in bulk materials. Analysis of the diffractograms provides quantitative details such as particle size, strain, the degree of ordering, texturing and superstructures. Low-angle (or low *q*) methods are used to probe longer periodicities such as multilayers and superstructures. The role of X-ray diffraction in the characterization of thin epitaxial films, multilayers and superlattices have been reviewed in various studies [174,175].

The study of structures in which the long-range periodicity is lost is referred to as scattering techniques, such as X-ray scattering (XRS). These are relevant for the study of amorphous materials [173], providing structural details in terms of pair correlation functions. At low angles, the X-ray scattering signal is proportional to the Fourier transform of the electron density [173].

The resonant X-ray diffraction and reflectivity (R-XRD and R-XRR) techniques exploit the rapid changes of atomic scattering factors close to an atomic absorption edge (anomalous scattering factors) to add a further degree of freedom to the techniques [176]. They combine element-specific information from X-ray absorption spectroscopy to Bragg diffraction and reflectivity to provide elemental selectivity diffractometric information [177,178] with the additional possibility to achieve elemental selective magnetic information [179,180].

### 3.1. Resonant X-ray Scattering (R-XRS)

The basis of the resonant X-ray scattering (XRS) technique (or anomalous X-ray scattering) is the anomalous scattering effect: the generalized scattering factors of a single atom can be described by [181].

(2) $$f(q,E)=f\left(q\right)+f^{\prime }\left(E\right)+if^{\prime \prime }\left(E\right)$$

where $f\left(q\right)$ is the Thomson scattering factor or atomic form factor (it is the Fourier transform of the atomic electron density), with $f\left(0\right)=Z$, and decays as a function of q, but it is independent of the incident photon energy *E*. The similar behavior of $f\left(q\right)$ for close *Z* elements, makes it difficult to distinguish the role of atoms having similar atomic number in the sample structure. $f^{\prime }\left(E\right)$ and $f^{\prime \prime }\left(E\right)$ are, respectively, the real and imaginary part of the anomalous scattering factor and depict a rapid variation in the proximities of an atomic absorption edge. Therefore, tuning the X-ray beam energy close to an absorption edge in the sample may change the contrast of specific atomic species and enhance the chemical sensitivity of the resonant scattering techniques, such as XRD and XRR. This allows for the recognition of the structural features ascribed to specific atoms in the sample structure. An important characteristic of resonant X-ray scattering techniques is the possibility of obtaining magnetic information as a counterpart of X-ray magnetic circular dichroism (XMCD). Historically, neutron scattering has been the magnetic scattering technique of choice for such investigations because X-ray’s sensitivity to magnetic structures is a second-order effect. But the improved beam quality at synchrotron radiation facilities and the possibility to tune the X-ray beam close to the absorption edges have enabled new types of magnetic scattering measurements [179,180] with advantages with respect to the neutron scattering of elemental selectivity, a wider *q* and higher spatial resolution, and the possibility to distinguish between spin and orbital contribution to the magnetic response [182].

Dealing with magnetic materials it is especially important to probe L_2,3_/M_4,5_ edges of transition/rare-earths metals because they involve transitions to 3d and 4f states respectively, which are involved in the magnetic response [183,184,185,186,187]. As an example of how it helps in thin-film characterization, we take an example of YMn_0.5_Fe_0.5_O_3_ epitaxial thin films [187]. After the successful deposition through MBE, the determination of the structure is the first prerequisite step, and identifying the relative arrangement of the Fe and Mn in the crystallographic sites is a relevant detail. By comparing diffractograms collected far from and close to the Fe/Mn absorption edge, an independent dataset is obtained with different contrast for Mn^3+^ and Fe^3+^ ions, allowing for a conclusion about their crystallographic positions. Noticeably neutron diffraction is also effective in distinguishing between Fe and Mn owing to their different scattering lengths. However, the quantity of samples in the epitaxial thin film is often low for neutron diffraction experiment.

### 3.2. High-Resolution X-ray Diffraction (HRXRD)

XRD is a fundamental non-destructive, quantitative technique for characterizing the atomic-scale structure and lattice defects of crystalline materials. When applied to thin films and heterostructures, high-resolution XRD provides detailed information about textures, phases, strain state, crystallinity and interface effects [89,90,188,189]. This makes it an essential tool for fundamental studies and the process development of advanced thin-film materials, devices and interfaces.

**Figure 5:**
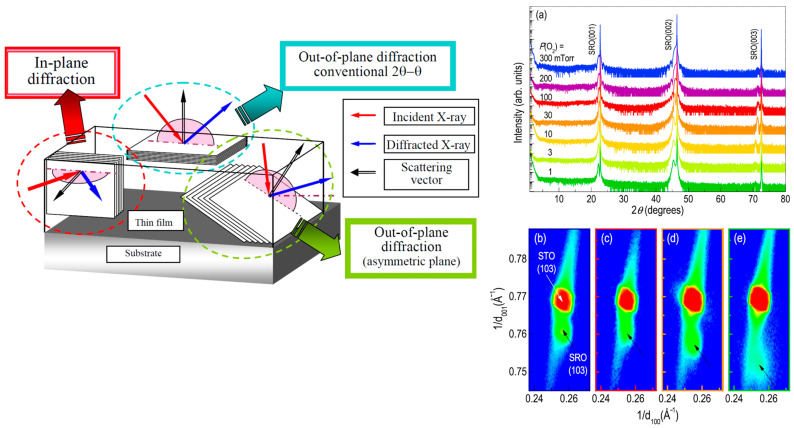
(**Left**) A schematic diagram of the geometries of thin-film high-resolution X-ray diffraction measurement, showing relations between the lattice planes of a thin-film sample and X-ray geometries. Adapted from Ref. [190]. (**Right**) High-quality heteroepitaxial SrRuO_3_ thin films with varying P(O_2_). (**a**) XRD θ-2θ scans for epitaxial of SrRuO_3_ shifts to a lower angle, indicating an increase in the c-axis lattice constant. XRD reciprocal space mapping of the SrRuO_3_ thin film grown at P(O_2_) = (**b**) 300, (**c**) 100, (**d**) 30, and (**e**) 1 mTorr around the (103) Bragg reflection of the SrTiO_3_ substrate, which shows a coherently strained film with the same in-plane lattice constant, respectively. Adapted from Ref. [191].

By exploiting high-brightness X-ray sources, such as those available at synchrotron radiation facilities, coupled with high-precision optics and detectors, angular resolutions down to the arc-second scale can be routinely achieved. This enables measurements of extremely small lattice spacings and subtle peak shifts related to strain and composition. High-resolution rocking curve analyses and reciprocal space maps (RSM) probe mosaicity, tilting, relaxation and defects within films and at buried interfaces (Figure 5 as an example). Kinematic scattering models can determine film thickness, roughness, density and buried interfacial structure with sub-nanometer precision.

The magnetoelastic nature of most magnetic materials means that HRXRD can provide critical insights into the intimate connection between magnetic properties and structural order. Reciprocal space mapping reveals in-plane and out-of-plane strains that influence magnetic anisotropy, which is relevant for specific applications of thin magnetic films and important for the fundamental comprehension of their magnetic–structural relationship.

At synchrotron radiation facilities, resonant (or anomalous) X-ray diffraction (R-XRD) exploits the rapid variation of the atomic scattering factor close to an atomic absorption edge in the sample to add chemical sensitivity and allows for the element-specific probing of magnetic sublattices to elucidate complex spin structures. Grazing incidence X-ray diffraction (GIXRD) geometries enhance surface sensitivity and allow for the depth-resolved profiling of structure and strain gradients in layered systems.

### 3.3. High-Resolution Crystal Truncation Rod Scattering (HRCTRS)

Crystal truncation rod (CTR) scattering, an extension of conventional X-ray diffraction, as a valuable and distinctive tool for determining the atomic structure of surface and interfacial systems [192,193]. In short, the CTR technique can provide full information about the atomic constituents and positions of surface and near-surface structures. Importantly, the technique is depth-sensitive and can be applied to heteroepitaxial systems with ultrathin layers (in the order of 10 nm or less) and even buried interfaces, which are often inaccessible by other surface characterization tools [194]. In terms of the spatial resolution of atomic positions, high-quality CTR datasets measured at synchrotron X-ray sources routinely provide layer-resolved atomic positions with precisions in the range of 1–10 pm in the surface-normal direction.

### 3.4. Grazing Incidence X-ray Diffraction (GIXRD)

It is important to note that for thin films, dimensionality plays an important role. As a matter of fact, due to the thinness of the film, the scattering volume is normally very small, which results in weak diffracted intensities with respect to the signal coming from the substrate. Surface-sensitive methods are achieved, reducing the angle of incidence of the beam (Figure 4b,c). This increases the effective radiation path in the surface layers while reducing the radiation transmitted in the deep layers. These methods are generally named grazing incidence X-ray diffraction (GIXRD) [195,196]. Geometrically, parallel X-rays are incident to the thin films or multilayers at fixed grazing angles (few degrees or less, generally limited by beam size and sample length), and the diffracted beam are detected by the detector by scanning along 2θ of the specimen. This allows for the control of the penetration depth of the X-rays in the sample, thus minimizing the contribution of the substrate. Among are advantages of GIXRD for thin-film investigations are as follows: (i) The enhanced surface sensitivity. GIXRD provides enhanced surface sensitivity compared to conventional XRD. Reducing the angle of incidence α (Figure 4b,c) X-rays penetrate the film at a shallow angle and interact primarily with the near-surface region while the intensity of primary beam is progressively attenuated by the effective X-ray path inside the sample. This allows for a more accurate characterization of the surface structure, surface roughness and surface-related phenomena in thin films. (ii) The reduction in substrate contributions in the diffraction patterns. In thin-film samples, the XRD signal from the substrate significantly hinders the thin-film signal and complicate the analysis. GIXRD allows for the minimization of the contribution from the substrate by utilizing a grazing angle, which reduces the intensity of substrate-related diffraction peaks and enables a clearer interpretation of the thin film’s diffraction pattern, facilitating the extraction of specific information about the film itself. (iii) Exploiting micro- and sub-micrometric highly collimated beams. Like on modern synchrotron radiation-dedicated beamlines, smaller angles can reach, allowing for the probing of crystallographic orientation, lattice parameters and phases in ultrathin films [94,197]. (iv) Analysis of preferred orientation. Thin films often exhibit preferred orientation, where structural features (stress, crystallite orientation and phases) tend to be different, i.e., parallel and perpendicular, to the surface. Owing to the sensitivity of diffraction to structural features parallel to the scattering vector, the in-plane and out-of-plane geometries (Figure 4b,c) allow for the selective probing of preferred orientation effects in-plane and out-of-plane. By varying the grazing angle and analyzing the corresponding diffraction patterns, researchers can quantify and understand the extent and nature of preferred orientation in thin films [31,93,198]. In situ and time-resolved studies can be performed during thin-film growth or the tuning of the environmental conditions (temperature, pressure and reactive atmosphere), providing real-time information on the structural evolution of the film. By monitoring the changes in the diffraction patterns during film deposition or under external stimuli, researchers can investigate the growth kinetics, phase transformations, and dynamic processes occurring in thin films [199,200].

In summary, GIXRD is preferred over conventional XRD for thin-film characterization due to its enhanced surface sensitivity, reduced substrate contributions, capability to analyze ultrathin films, ability to study preferred orientation effects, and its potential for in situ and time-resolved studies. It offers valuable insights into the surface structure, surface-related phenomena, crystallographic orientation, and phase transitions in thin films, contributing to a comprehensive understanding of their structural properties and behavior.

### 3.5. Grazing Incidence Small-Angle Scattering (GISAXS) 

As highlighted above, at low angles (low *q* values), the scattering signal become sensitive to the medium-range order (nm range), therefore small-angle scattering techniques, like small-angle neutron scattering (SANS) and small-angle X-ray scattering (SAXS), are powerful methods to characterize the structures of nanostructures (macromolecules, nanoparticles and mesoscale structures) in the 10^0^–10^2^ nm size scale. By measuring the scattering intensity as a function of the scattering angle, information about the size, shape, internal structure and interactions of the particles can be determined. As for XRD, in order to investigate nanostructures and surface morphology of thin films and layered materials, grazing incidence geometry can be used to enhance the surface signal with respect to bulk and substrates [95,96,201].

GISAXS is used to investigate the nanostructures and surface morphology of thin films. Being sensitive to nanoscale variations of electron densities, GISAXS provides information about the size, shape, spatial arrangement and interactions of nanoscale features close to the surface regions of the samples. GISAXS can reveal nanosized structures, pores, phase separation, self-assembly and more nanostructured features. The GISAXS techniques are particularly suitable for studying thin films with nanoscale features, including ultrathin films. It provides complementary information with respect to GIXRD about the medium-range order, inhomogeneities, phase aggregation, amorphous phase and disorder. The basic difference between the two techniques has been listed in Table 5. Combining GIXRD and GISAXS techniques is often suitable to gain a more complete understanding of the film’s properties. Exploiting the resonant effect, that is, tuning the X-ray beam close to an atomic absorption edge in the sample, allows element-specific details to be added.

### 3.6. X-ray Reflectivity (XRR)

**Figure 6:**
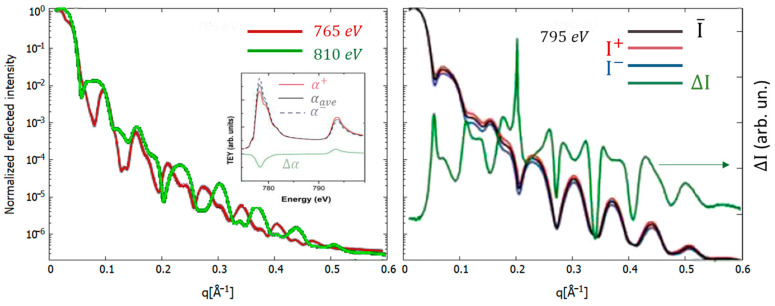
Resonant effect in soft X-ray reflectivity data from MgO/Co/MgO trilayer. Left panel: XRR curves measured well below (765 eV) and above (810 eV) the Co L_II,III_ edges. In the inset: the Co L_II,III_-edges absorption spectra measured with left ($\alpha^{-}$) and right ($\alpha^{+}$) hand circularly polarized beam, along with their average ($\alpha_{ave}$) and difference (ΔI, down shifted for clarity). Right panel: XMCD effect on XRR: the reflectivity curves measured with opposite ($I^{-}$, $I^{+}$) circularly polarized X-ray beam at the maximum of Co-L_III_ edge. The average XRR ($\bar{I}=(I^{+}+I^{-})/2$) and normalized difference ($\Delta I={(I}^{+}-I^{-})/{(I}^{+}+I^{-})$) are shown [203] ΔI scale is linear (right arrow). In the inset the Co L-edges.

X-ray (or neutron) reflectivity (XRR and NR) is a powerful technique for investigating thin film and multilayer structures in a non-destructive way [80,81,82,202]. The basic principle of XRR is to measure the specular reflected X-ray beam intensity from a flat surface or layered system. XRR utilizes the reflection of low-angle X-rays in the 10^−1^–10^0^ nm^−1^ q range. In dealing with thin films and multilayers, the interference between X-rays reflected at each interface gives rise to oscillations in the reflection intensity as a function of the exchanged momentum vector *q* (Kiessig fringes). The period and features of these oscillations can be used to infer morphological and structural details about the layered samples: layer thicknesses, interlayer roughness, electron densities and their contrasts, refractive indices (which depend on layer composition via the atomic numbers and complex atomic form factor) and depth-dependent composition gradients. As shown in Figure 6, the period of interference fringes is related to the film thickness *d* (or multilayer periodicity), the amplitude of the oscillations is higher for higher electron density contrast between the layers, and the decay rate of Kiessig fringe intensity is related to the surface/interface roughness.

XRR modeling can precisely determine thickness and roughness at buried interfaces within multilayer stacks. In situ XRR during deposition or processing reveals growth kinetics and thermal evolution. With atomic-scale sensitivity to vertical structures and interfaces, XRR is an essential characterization tool for elucidating the relationships between film morphology (layer thickness and composition, interface roughness and degree of intermixing) and macroscopic properties. XRR analysis provides information on the following: (i) film thickness, which is related to the periodicity of intensity fringes as a function of the angle of reflection θ, with thinner/thicker layers giving longer/sharper fringes. In dealing with complex structure with several layers and periodic multilayers, Parratt formalism [202] allows multiple interferences to be considered. (ii) Accurate electron density and composition profile across the film and multi-layer systems can be obtained by analyzing the shape and intensity of the X-ray reflectivity curve. Variations in the electron density reveal compositional changes or the presence of interfacial layers, such as oxides or interdiffusion zones. (iii) Surface/interfaces roughness affects the intensity and width of the reflection fringes and can be calculated, modeling the experimental as root-mean-square roughness. Off-specular reflectivity (rocking curves) provides information about roughness correlation length. Effects of interdiffusion and the formation of interfacial layers can be quantified as well. (iv) In situ and real-time XRR experiments, available at high brilliance synchrotron radiation facilities, allow for the probing of the film during deposition processes and can be used to understand the kinetics response of the films/layers to externa stimuli such as temperature, pressure and strain.

The ability to tune the X-ray beam close to the atomic absorption edge available at synchrotron radiation facilities adds the possibility of exploiting the resonance effect for XRR (R-XRR) as well, adding a valuable degree of freedom useful to localize morphological features associated with specific atomic species in the sample. In Figure 6, the R-XRR effect observed measuring the XRR at different energies below, at and above the Co-L_2,3_ edges in a MgO/Co/MgO trilayer system is evident [203]. The L_2,3_ absorption edges of transition metals (and M_4,5_ of rare earths) are associated with excitations of the core electrons in the 3d levels (4f of rare earths). Since the occupancy of the *d*(*f*) states is different for spin-up and spin-down states, in the case of a magnetic ion the absorption probability of circularly polarized X-rays can be different depending on the helicity direction of the probe, giving rise to an effect of X-ray magnetic circular dichroism (XMCD, see below), which can be used as an additional contrast factor in reflectivity measurements, to study details of the morphology of magnetically active layers. An example is presented in Figure 6 (right panel), in which the effect of XMCD provide differences in the reflected intensities measured with left ($I^{-}$)- and right ($I^{+}$)-hand-polarized beams with a large XRR difference, where $\Delta I=(I^{+}-I^{-})/(I^{+}+I^{-})$. The contemporary analysis of several XRR patterns along with XMCD spectra permitted the authors to obtain an accurate characterization of the multilayer morphology, including the distribution of magnetic layers [87,203,204,205]

Overall, X-ray reflectivity is a valuable technique for interface characterization in thin films, providing crucial information about film thickness, density, composition, roughness, and interfacial structure. It is widely used in materials science, surface science, and thin-film technology for understanding and optimizing thin-film properties and performance. Table 6 shows an overview of the different structural information that can be derived for a variety of thin films from the combination of XRPD, HRXRD, GIXRD, GISAXS and XRR techniques.

### 3.7. Grazing Incidence X-ray Fluorescence (GIXRF)

The basic concept of grazing incidence X-ray fluorescence (GIXRF) technique is to measure the fluorescence signal emitted by the elements present in the sample as a function of the angle of incidence of the X-rays. In 1991, De Boer presented a theoretical formulation for the fluorescence emitted (GIXRF) from layered samples based on the calculation of the derivative of the Poynting vector through the determination of the reflection and transmission coefficients [122]. GIXRF allows for the evaluation of the atomic distribution through the layer depth profile. A combination of X-ray reflectometry (XRR) and grazing incidence X-ray fluorescence (GIXRF) has become a strong tool in the analysis of thin films. Both use similar measurement procedures, and the same fundamental physical principles can be used to analyze the data [78,123]. The major difference comes from the fact that GIXRF evaluates the atomic distribution through the layer depth profile by measuring the X-ray fluorescence intensities of the involved elements as a function of the incident angle. Noticeably XRR is poorly sensitive to stacked layers with similar electron densities, thus GIXRS may provide complementary information and can be conveniently used for the elemental depth profiling of thin layers and in the distinct analysis of a buried layer or an interface.

### 3.8. X-ray Standing Wave Geometry (XSW)

**Figure 7:**
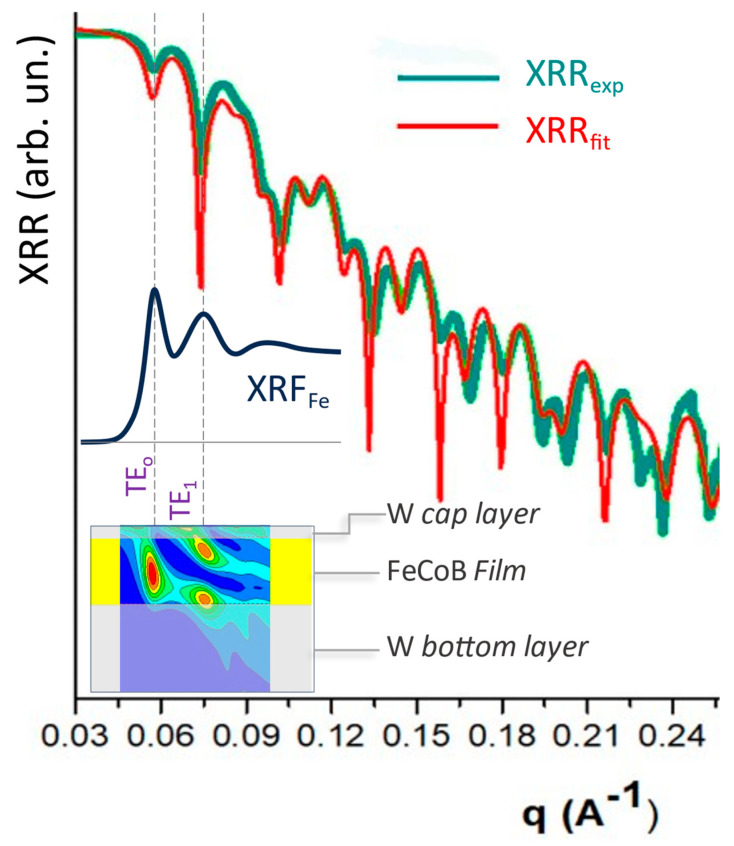
Example of XSW measurements (adapted from Ref. [108]) experimental XRR data (XRR_exp_) and model (XRR_fit_) curve from a waveguide made by a thin FeCoB film between two W layers. The measured Fe XRF signal is shown (middle curve). The X-ray intensity field inside the waveguide, as a function of the exchanged momentum q, is presented at the bottom. The antinodes of XSW modes TE_o_ and TE_1_ (red regions in the bottom schema) are located at the center and borders of the FeCoB film, respectively.

The concept of X-ray standing wave (XSW) technique was first mentioned by B. W. Batterman in the 1960s [206]. It is based on the X-ray interference method where two coherent X-ray beams reflected by the superposed layers of a stratified sample, generating variable electric field intensity fluctuations perpendicular to the reflecting planes [124,207,208], and a periodic electric field is established when the Bragg conditions (Equation (1)) are fulfilled and, like mechanical standing waves, it generates nodes and antinodes. An X-ray standing wave field is two-dimensional, and in the nodal planes, the amplitude of the electric field is suppressed, while in the anti-nodal planes, it is enhanced. The feature of the X-ray standing waves makes them quite suitable for the analysis of periodic layered structures, where the electric field is formed under Bragg conditions. By varying the parameters (wavelength and angle) of the X-ray beams, one may modulate the position of the anti-nodal planes of the electric field to improve the profiling of ultra-shallow depth distributions [78,123]. Combining XSW with XRF or X-ray absorption spectroscopies (Figure 7) can provide depth-selective information about composition (XRF), local atomic structure (XAFS) and magnetic (XMCD) information.

The application of XSW techniques requires the special preparation of the samples. The layer of interest can be realized as a waveguide owing to the refraction index for X-rays less than unit, and an X-ray waveguide is realized, sandwiching a light (low electronic density) layer between two heavier (high electronic density) layers [209,210]. Higher resolution can be achieved by growing the layer of interest over a multilayer, acting as a Bragg mirror [211]. Special geometry can be realized by growing the layer of interest on a wedge geometry over a multilayer mirror [109].

### 3.9. Low-Energy Electron Diffraction (LEED)

This technique is an electron analog of XRD that can provide the crystal structure over the outer 2–5 nm of a solid’s surface. This surface specificity arises from the fact that unlike photons, low-energy electrons (20–200 eV) can only travel a very short distance within a solid before suffering elastic and inelastic collisions, whereupon they lose energy. Electrons are directed normally to the surface, and the backscattered signal (elastically scattered) is recorded. This displays an XRD-like diffraction pattern. As electrons are used, high vacuum (HV) and clean surfaces are required.

### 3.10. Reflection High-Energy Electron Diffraction (RHEED)

**Figure 8:**
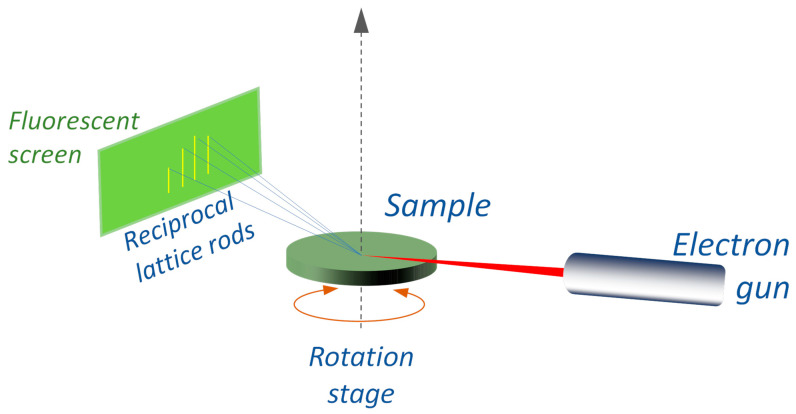
Schematic diagram of RHEED setup.

It is an electron analog of GIXRD that can provide the crystal structure of the outer 1–10 nm by directing a high-energy electron beam (∼10–100 keV) at some glancing angle and measuring the forward scattered signal (Figure 8). The lattice order is revealed in the reflected beam because of the diffraction induced by the surface crystalline planes. Although LEED provides better diffraction patterns, RHEED allows for improved sample access (which is needed in epitaxial growth studies).

### 3.11. Polarized Neutron Reflectometry (PNR)

It is a powerful technique for the structural and magnetic characterization of thin films and multilayers, which enables depth-resolved magnetization measurements in films with characteristic thicknesses from 10 to 5000 Å [97,98,102,103,104,212]. The uniqueness of accessing magnetism comes from the fact that neutrons possess a magnetic moment that allows them to interact with the magnetic moments in a sample. Also, the wavelengths are comparable to interatomic distances, allowing for diffraction measurements that provide structural information especially for light elements.

The sensitivity of the NR technique to interfaces is because the projection of the neutron wavelength normal to the surface matches the thickness of thin films, and the neutron wave function becomes strongly distorted near interfaces when the neutron encounters a potential step [101,212,213]. NR probes the nuclear scattering length density (NSLD) profile perpendicular to the surfaces and interfaces (which contrasts with the electron scattering length density (ESLD) in XRR), the interface roughness and the interface morphology [100]. Thus, the technique allows for the investigation of buried layers and interfaces in thin films and can also be used for in situ measurements.

**Figure 9:**
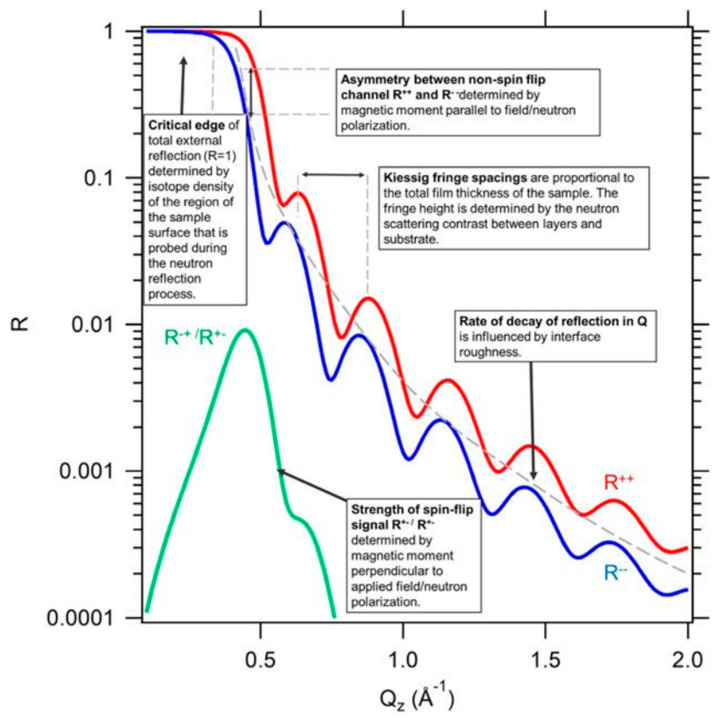
A simplified guide to decoding a neutron reflectometry profile showing how layer thickness, chemical composition and magnetization can affect the observed signal. The example is for a 40 nm thick nickel film on a silicon substrate, with a magnetic moment of 0.6 μ_B_ per Ni atom, rotated at 45° to the in-plane applied field. Adapted from Ref. [104].

Speaking of the working technique, by polarizing the incoming neutron spin eigenstate and analyzing the reflected spin state, two types of neutron reflectivity can be obtained: the first one is the non-spin-flip (NSF), and the second one is the spin-flip (SF) reflectivity. For NSF neutron reflectivity (R++ and R−−), the spin polarizations are the same for the incoming and reflected neutrons (see Figure 9), and the component of the magnetization parallel or antiparallel to the neutron quantization direction can be determined. As for the SF neutron reflectivity (R+− and R−+), the reflected neutrons possess an opposite spin polarization with respect to the incoming neutrons. The component of magnetization perpendicular to the incident neutron quantization direction can thus be probed. Combining the results from NSF and SF neutron reflectivities allows the magnitude and the direction of the magnetic induction to be determined, which is apparently a quantitative depth-dependent vector magnetometer.

### 3.12. Resonant X-ray Coherent Diffractive Imaging (CDI)

**Figure 10:**
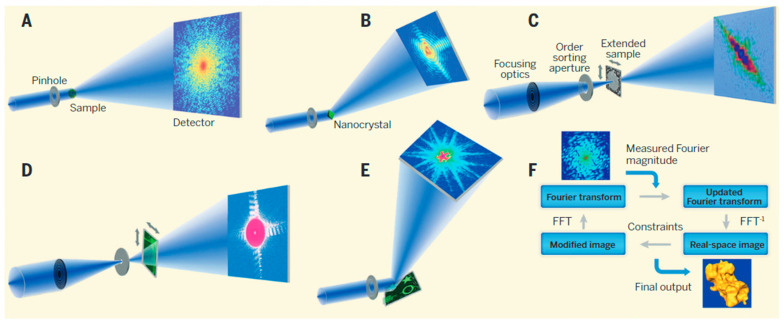
Illustration of (**A**) plane-wave CDI, (**B**) Bragg CDI, (**C**) ptychographic CDI, (**D**) Fresnel CDI, (**E**) reflection CDI, and (**F**) phase retrieval algorithms iterating back and forth between real and reciprocal space. In each iteration, various constraints, including support, positivity (i.e., electron density cannot be negative) and partially overlapping regions, are enforced in real space, while the measured Fourier magnitude is updated in reciprocal space. Usually, after hundreds to thousands of iterations, the correct phase information can be obtained. Adapted from Ref. [107].

**Figure 11:**
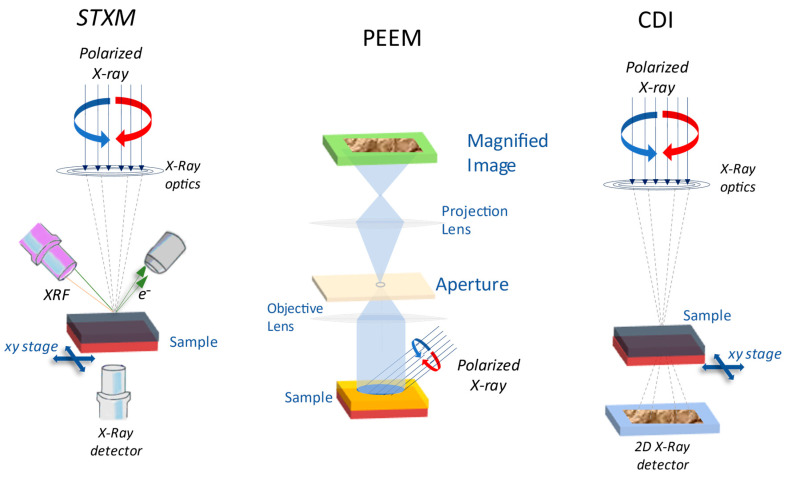
XMCD-based microscopy techniques [221]. Contrast is obtained by comparing the images collected using left (blue) and right (red) circularly polarized X-rays, allowing detailed and chemically selective information on the magnetic order in the samples to be obtained.

Coherent diffractive imaging (CDI) is an alternative promising “lensless”, aberration-free, high-resolution three-dimensional imaging technique (Figure 10) widely used in the physical and biological sciences [105,106,107,214,215,216,217,218]. The diffraction pattern is formed by scattering a coherent X-ray beam from a sample. The recorded pattern is then used to reconstruct the sample details via an iterative feedback algorithm [219,220]. In CDI, the spatial resolution is not limited by the quality of the optical elements but by the highest spatial frequencies measured in the X-ray diffraction pattern, and the various modes are shown in Figure 10. The main advantage of using this technique is constructing 3D images of defect structures in magnetic multilayers [221,222], the tomographic imaging of misfit dislocations at interfaces [223], free of the thin-film elastic relaxation processes, which distort the images obtained by transmission electron microscopy and imaging strain on the nanometer scale in three dimensions [214,224]. A comparison of CDI method with PEEM and STXM has been illustrated in Figure 11.

## 4. Spectrometric Techniques

A spectrum reports the intensity absorbed, emitted or scattered by a material as a function of the wavelength or frequency of the probe, i.e., electromagnetic radiation or particles. The analysis of the spectra can provide a wealth of information about the properties and characteristics of materials, including chemical composition, electronic structure and transport properties, atomic structure static and dynamics, and magnetic properties. In the following, we focus on their application to thin magnetic films and layers.

### 4.1. X-ray Absorption Spectroscopy (XAS)

X-ray absorption spectroscopy (XAS) is chemically selective, local-sensitive techniques that exploit tunable synchrotron X-ray sources to excite and probe atomic absorption edges providing element-specific information about local coordination chemistry, atomic structure, and electronic and even magnetic state. The X-ray absorption fine structure (XAFS) signal refers to the tiny oscillations appearing at energies near and above a core absorption edge of a not isolated atom. This corresponds to the modulation of an atom’s X-ray absorption probability due to the chemical and physical state of the atom. The success of XAFS in any field requiring the study of matter and materials stems from its very wide versatility [225,226] It is applicable to materials in any aggregation state (gases, liquids and solids), measurable from bulk to the highest diluted samples (micro- and nano-molar), in any form (bulk, surfaces, layered structures, quantum and nano structures), and allows for fast (minute-seconds range) and ultrafast (pico-seconds scale) data collection for in situ studies. Standard measurements (generally) require a relatively simple experimental setup and easy data collection.

X-ray Absorption Near-Edge Structure (XANES) and Extended X-ray Absorption Fine Structure (EXAFS), modulations in the X-ray absorption coefficient above an atomic edge provide complementary information: the analysis of XANES features (full multiple-scattering region (FMS)) allows for the description of the absorbed ionization state and the symmetry of the coordination environment, and the analysis of EXAFS features allows for the description of the radial distribution function around the average absorber, providing details about the average neighbor distances and the mean-square relative displacement. A specific feature of XAFS, which is different from any other scattering techniques, is its sensitivity to multiple scattering contributions, providing topological information about the local structure around the absorber and details about the many-body distribution functions [227,228]. For thin-film materials, XAS offers exceptional chemical sensitivity for characterizing the structure and bonding of interfaces and buried layers, including alloy configurations, interface reactions and depth gradients.

Additional XAS-related techniques provide further capabilities: X-ray linear dichroism exploits the linear polarized synchrotron radiation to provide some directional sensitivity (polarized XAS) to probe structural anisotropies and bond orientation details. (SW-XAFS) allows for depth selectivity studies to specifically probe atomic structure and electronic properties in thin films and interfaces. The X-ray magnetic circular dichroism (XMCD) can provide element-selective magnetic state information with the special possibility to disentangle orbital and spin contributions to the atomic magnetic moments. Resonant Inelastic X-ray Scattering (RIXS) is used to achieve excited state spectroscopy.

For thin and ultrathin films, standard X-ray diffraction techniques face challenges in accurately determining structure and phase. At nanometer scales, intrinsic disorders from polycrystallinity along with Debye–Sherrer peak broadening can significantly broaden diffraction peaks, obscuring subtle features. Ultrathin layers often exhibit strain, which shifts peak positions. Further complexity arises when interference between interfaces can cause extensive peak overlap. Therefore, due to its local structure sensitivity and chemical specificity, XAS has become an essential tool for the advanced understanding of thin-film properties. As an example, the Fe- and Co-K edges in XAFS analysis allow for the distinguishing of local-order differences between Fe and Co in thin FeCoB magnetic films [108]. XAS has proven to be a suitable technique to investigate the local structure mechanisms related to peculiar magnetic properties in thin films. The technical improvements in beam stability, optics, and detectors at synchrotron radiation facilities have enabled highly stable and reproducible X-ray absorption fine structure (XAFS) measurements. This enhanced precision allows even subtle atomic displacements induced by magnetostriction in FeCo ferromagnetic films to be detected at an fm resolution [229]

The versatility of XAS-based techniques has allowed for the development of sophisticated and original measurement methods specifically suited to the study of thin films, as we will see below.

### 4.2. Standing Wave X-ray Absorption Fine Structure (SW-XAFS)

**Figure 12:**
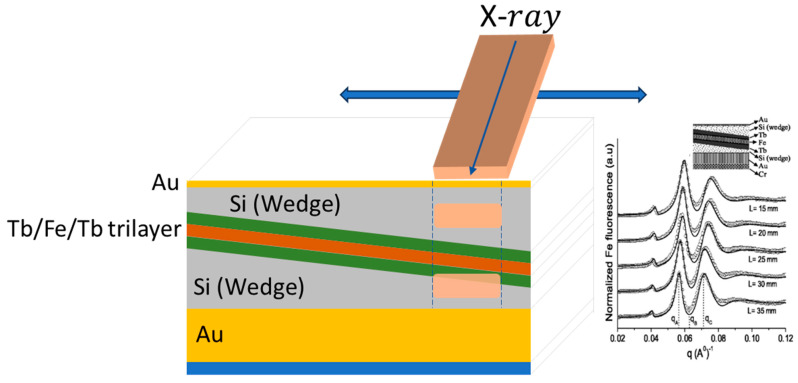
Tb/Fe/Tb trilayer was grown obliquely within an Au/Si/Au waveguide, the TE_1_ antinodes are schematically shown in the schema (rose rectangles). Moving the X-ray spot along the wedge allows for the tuning of the SW antinodes with different portions of the trilayer. Adapted from Ref. [109].

Coupling XAFS with XSW geometry is a way to provide depth selectivity insights about the structural and chemical properties of thin films and interfaces [109,110,209,210,230,231,232]. By taking advantage of the interference between the incident and reflected X-rays, standing wave XAFS offers specific advantages for thin-film characterization. Depth sensitive information can be gathered coupling the specific characteristics of XAFS with the possibility of localizing the SW radiation field across the film thickness. A fixed-q setup is required to scan the X-ray energy across the absorption edge of a specific element in the film, while keeping the SW field tuned to have antinodes at a specific depth in the film. This depth sensitivity allows for the probing of the structural, electronic and chemical properties of thin films at different depths [209,210] and can be useful to specifically probe thin-film interfaces with the substrate or other layers, which can significantly impact their macroscopic properties. Specifically wedge-shaped sample has been used [110] to specially probe the local structure characteristics of a Tb/Fe/Tb trilayer, as shown in Figure 12. Fe-K edge polarized SW-XAFS measurements provided sufficient depth resolution to differentiate the center of the Fe layer from the interfacial region, and by exploiting the directional sensitivity of polarized measurements, it has been proved that Fe–Tb bonds in the interfacial region lie preferentially normal to the film plane and are likely responsible for the PMA of Fe/Tb films.

SW-XAFS can be performed in situ during thin-film growth or under various environmental conditions, enabling the study of the evolution of the film structure and chemical properties in real time. By monitoring changes in the XAFS spectra during film growth or under external stimuli, researchers can gain insights into the kinetics, phase transformations and dynamic processes occurring in thin films.

In summary, standing wave XAFS offers depth sensitivity, interface characterization capabilities, and element-specific analysis, making it a valuable technique for understanding the structural and chemical properties of thin films. It provides information about the local atomic structure, electronic states and element-specific coordination in different regions of the film, contributing to a comprehensive understanding of the microstructural origin of the magnetic properties of thin-film systems.

### 4.3. Polarized X-ray Absorption Fine Structure (P-XAFS)

Owing to the preferred photoelectron emission along the electric field direction and the natural linear polarization of synchrotron radiation, XAS techniques can provide directional sensitivity along the X-ray beam polarization. Polarized XAFS (P-XAFS) is valuable as it offers additional insights into the orientation and symmetry of atomic bonds within the thin film and at the interfaces [231,233,234]. The P-XAFS measurements are carried out by orienting the sample with respect to the X-ray beam polarization direction. This allows for probing of bond orientation and local structure anisotropy. By using linearly polarized X-rays, researchers can selectively excite and probe specific atomic bonds along the X-ray polarization direction and detect anisotropies that can be structural (bond length) or compositional (preferred neighbors). This information is especially relevant for ultrathin films and interfaces where the layer becomes intrinsically anisotropic. Local structure and compositional anisotropies are often related to anisotropic macroscopic response as PMA. As examples P-XAFS is particularly suited to probe the microstructural mechanisms at the origin of PMA in thin films. As an example, it has been demonstrated that the PMA in epitaxial fcc CoPt_3_ (111) films stems from the existence of anisotropic local ordering characterized by preferential CoCo pairs in the film plane, balanced by preferential CoPt pairs out of the film plane [111]. The analysis of Co-K edge P-XAFS proved the magnetic response of Co films intercalated between graphene and Ir(111) is related to the local anisotropy, consisting of stretched Co-Co bonds in the film plane and compressed outside the plane [232].

### 4.4. X-ray Magnetic Circular Dichroism (XMCD)

**Figure 13:**
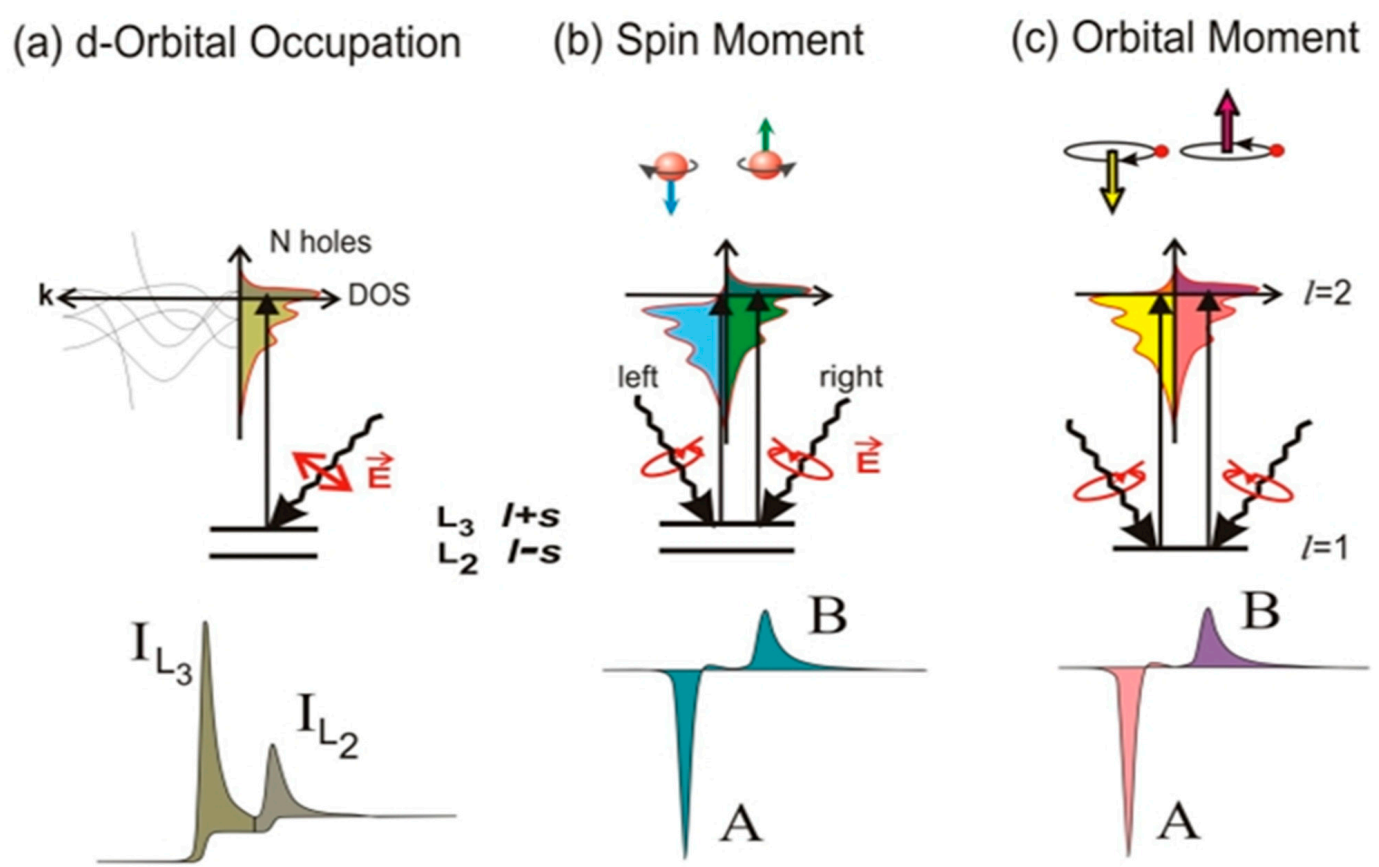
Electronic transitions in conventional L-edge X-ray absorption (**a**) (linearly polarized X-rays), and X-ray magnetic circular X-ray dichroism (**b**,**c**), illustrated in a one-electron model. The transitions occur from the spin-orbit split 2p core shell to empty conduction band states. For unconventional X-ray absorption, the total transition intensity of the two peaks is proportional to the number of d holes (first sum rule). By the use of circularly polarized X-rays, the spin moment (**b**) and orbital moment (**c**) can be determined from linear combinations of the dichroic difference intensities A and B, according to other sum rules. Adapted from https://www-ssrl.slac.stanford.edu/stohr/xmcd.htm (accessed on 18 November 2023).

XMCD arises from the differential absorption of left- and right-handed circularly polarized X-rays by magnetic elements due to their spin and orbital magnetic moments (Figure 13). By tuning the X-ray energy near an absorption edge of a specific magnetic element, XMCD can be maximized, providing a magnetic contrast that allows for the detection and analysis of the magnetic properties of thin films [113,114,235]. XMCD measurements provides element-specific information about the magnetization direction and magnetic moments, facilitating the study of the magnetization behavior and interactions of different elements within the film. It also enables the investigation of the magnetic depth profiles, including interfacial magnetic effects, layer-specific magnetization, magnetic domain structures and magnetic anisotropy.

### 4.5. X-ray Photoemission Spectroscopy

X-ray photoelectron spectroscopy (XPS) is an essential technique for investigating the surface and near-surface chemistry as well as the electronic structure of materials. In XPS, a sample surface is irradiated with monochromatic X-rays, causing photoelectrons to be emitted from core atomic levels. The main photoemission process involves the core-electron binding energy, $E_{B}$, and the material work function, $\varphi_{s}$, so that the photoemitted kinetic energy, $E_{k}$, is related to X-ray beam energy, $E_{x-ray}$, by the equation $E_{x-ray}=E_{B}+\varphi_{s}+E_{k}$ [236,237]. By analyzing the kinetic energy and number of the emitted photoelectrons using an electron energy analyzer, the characteristic core-level binding energies and elemental composition can be determined. Shifts in the binding energies provide chemical specificity, allowing different oxidation states and chemical environments to be distinguished.

**Figure 14:**
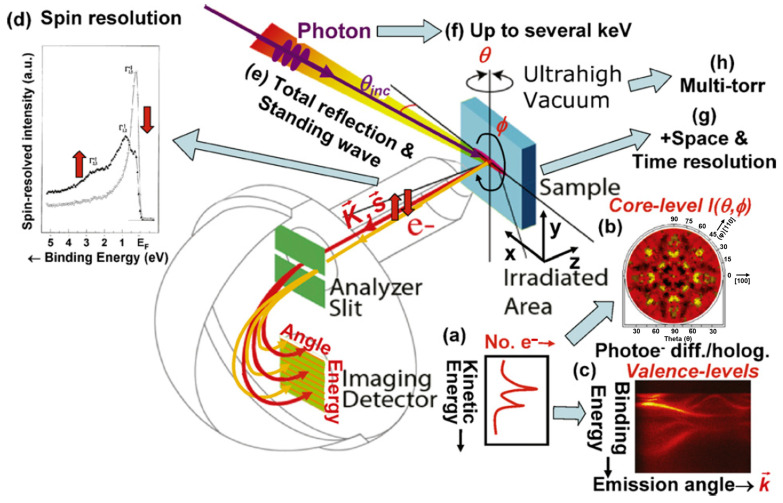
Illustration of different information obtained from XPS. Adapted from https://www.issp.u-tokyo.ac.jp/public/optics/LectureNote_IM2.pdf (accessed on 18 November 2023). (**a**) Photoelectron spectrum plotted against kinetic energy versus total counts of electrons, (**b**) core level photoelectron diffraction, (**c**) Probing of valence band vs *k,* (**d**) spin-resolved spectra, (**e**) tuning the photon energy to obtain total reflection or standing waves in the samples, (**f**) increasing of the photon energy to probe bulk electronic structure in the hard x-ray regime, provisions in XPS to conduct experiments in different conditions such as (**g**) space and time resolution and (**h**) high pressure conditions.

Dealing with thin films, XPS provides great sensitivity for analyzing the chemistry and physics of surfaces as well as close-to-surface interfaces and layers (see Figure 14). XPS chemical shifts reveal oxidation state, coordination and alloy configurations, while relative peak areas determine composition, including at buried interfaces. Angle-resolved XPS utilizes the photoelectron emission depth dependence to enable depth profiling and the analysis of composition gradients in layered structures. Additional capabilities are provided by ambient pressure XPS for in situ surface analysis and by XPS imaging for mapping chemical variations across a surface. XPS can also probe electron energy levels, valence bands, and the Fermi edge to reveal electronic structure and band alignment at thin-film interfaces. The Auger-photoelectron coincidence spectroscopy (APECS), i.e., the emission depth selectivity and the final spin-state selectivity, are specifically suited for studying magnetic films [238].

XPS has long been a widely used laboratory technique for surface and thin-film analysis using lab-based X-ray sources. However, the development of high-brightness synchrotron radiation light sources has opened many new possibilities for XPS. Some of the key advantages of synchrotron XPS over laboratory sources are the higher photon flux, leading to improved signal-to-noise and the ability to study lower concentrations and faster processes. Selectable and tunable photon energies allows for the optimization of photoionization cross-sections and electronic structure studies by resonant excitation. The ability to use micro-focused and nanoprobe beams allows for spatially resolved XPS at sub-micron-length scales. The pulsed time structure of synchrotron radiation, coupled with pump-probe setups, allows for time-resolved measurements on shorter time scales, down to picoseconds and below. X-ray beam polarization control (linear and circular) allows for the probing of excitation anisotropy and dichroic effects. These capabilities have driven innovation in XPS instrumentation at synchrotron facilities. Customized end-stations, electron analyzers, detectors and sample environments have extended the XPS technique into new regimes of elemental sensitivity, chemical imaging, depth profiling, real-time reactivity studies and electronic structure elucidation in physics, chemistry and materials science—especially for nanostructures, 2D materials and thin-film systems. While laboratory XPS is still widely used, synchrotron XPS is opening new scientific avenues in surface and interfacial research.

XPS has been widely used in concert with film synthesis to understand surface properties, interfacial charge transfer, electronic band alignment across a heterojunction, in-situ metal insulator transition, and non-idealities, such as intermixing and off-stoichiometry [115,116,117,118], either through core-level spectra or angle-resolved photoemission spectroscopy, which are discussed below.

a. Angle-resolved X-ray photoelectron spectroscopy (ARXPS)

**Figure 15:**
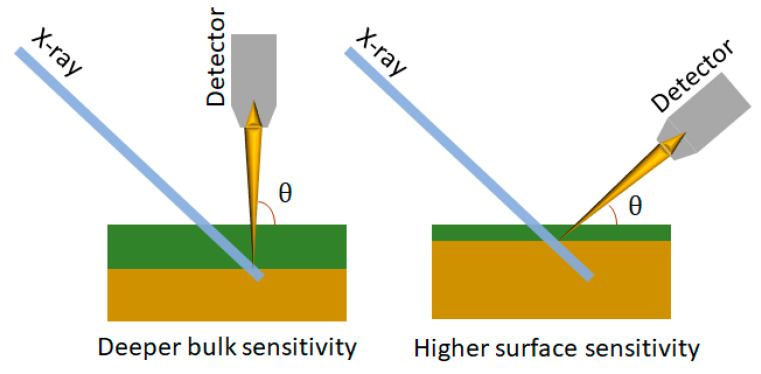
Schematic principle for ARXPS. Reducing the angle of the photoemitted electrons, θ, enhances the sensitivity to the surface layers.

It is a widely used non-destructive method to study the chemical composition and thickness of the outermost top layer of solid materials (some few nm) and therefore very attractive for the investigation of very thin layers [119,120,239,240,241]. ARXPS uses X-ray photons from a laboratory or a synchrotron source to eject electrons from a sample, analyzing their kinetic energy and emission angle to map out the binding energy and momentum of the electronic states, the angle-resolved XPS analysis is performed to obtain the depth selective information close to the film surface. The effective sampling depth is approximately t≃3λsin⁡θ, being a function of the photoelectron mean free path, λ, and the takeoff angle, θ, of the photoelectrons (Figure 15). At a lower takeoff angle mainly, the electrons emitted from the near surface region are detected, and the signals are predominantly from the surface of the sample. While raising the takeoff angles, the signals progressively contain information from deeper regions of the sample [241].

In the soft X-ray region, XAS data are usually recorded in electron-yield mode [226], in which the Auger electrons and secondary electrons, whose number is proportional to the X-ray absorption coefficient, are collected. Accordingly, the probing depth, λ, of the XAS data can be controlled by the electron emission angle, as shown in Figure 15. A specific setup providing sub nm resolution has been developed to specifically probe ultrathin magnetic films, exploiting XAFS and XMCD [242].

b. Spin-Polarized Photoemission Spectroscopy (SP-PES)

**Figure 16:**
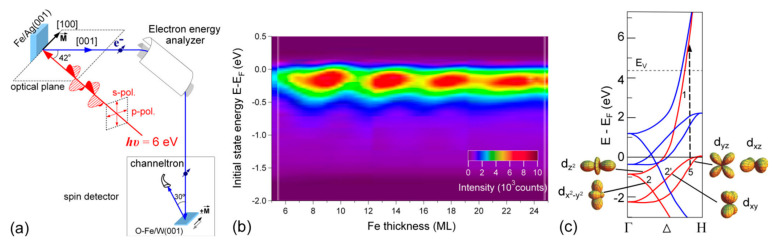
(**a**) Schematics of the spin-polarized PES experiment. (**b**) Thickness- and energy-dependent photoemission intensity distribution of Fe/Ag(001) at 300 K for s-polarized incident light, with energy hν = 6 eV. The intensity peaks below E_F_ correspond to the resonant transition depicted by a vertical arrow in the (**c**) spin-resolved bulk Fe(001) band structure. The red and blue curves represent the majority and minority spin-split bands respectively. Adapted from Ref. [245].

One of the most common methods for investigating the electronic and magnetic properties of two-dimensional systems is based on spin-resolved electron spectroscopies (Figure 16) [112]. The reason is found in the short inelastic mean free path of electrons in solids, which leads to an escape depth (ED) of only a few atomic layers for typical electron energies between 10 and 1000 eV. Such a low ED allows for a surface-sensitive analysis, while information on the magnetic character is provided by the spin resolution. Among spin-resolved electron spectroscopies, a prominent role is covered by those based on photoemission. In fact, spin-polarized photoemission spectroscopy (SP-PES), giving direct access to spin-resolved occupied electronic states, is widely used for investigating magnetic thin films and surfaces [74]. Moreover, the empty state region can be successfully probed by spin-polarized inverse photoemission spectroscopy (SP-IPES) [243,244]. The combined use of such spectroscopic techniques—without spin resolution—has already been shown to be an invaluable tool to derive the energetics of electronic states at interfaces.

### 4.6. Resonant Inelastic X-ray Scattering (RIXS)

**Figure 17:**
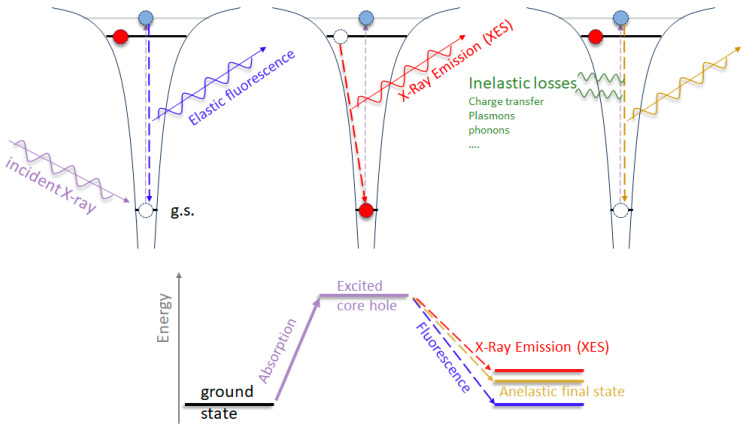
Top panel displays the schematic of photon-in–photon-out is the simplified atomic model of soft X-ray absorption spectroscopy (**left**), X-ray emission spectroscopy (**middle**), and resonant inelastic X-ray scattering (**right**). Lower panel displays the different states involved in the RIXS process upon energy scale (vertical).

Resonant Inelastic X-ray Scattering (RIXS) has emerged as a suitable and powerful chemically selective technique for studying the electronic structure of materials. RIXS is a photon -in and a photon-out process in which the energy of incident X-rays is tuned to a specific atomic absorption edge in the sample (chemical selectivity) to resonantly excite core electrons into unoccupied states, which then undergoes a radiative decay to the final state (Figure 17). Revealing the X-ray emission stemming from the relaxation of the excited state provides insights into the occupied and unoccupied electronic states which can be probed with high-energy resolution using specific spectrometers (references form SR beamline, such as ID26 at the ESRF). Due to the significant energy and momentum carried by X-ray photons, RIXS is capable of detecting charge, spin and orbital excitations in a momentum-resolved and chemically selective manner [125,126,127,246,247,248,249]. Owing to the high mean free path of incoming and fluorescence photons, RIXS is a bulk-sensitive method. However, for thin-film and interface studies, RIXS offers chemical selectivity, allowing for the probing of only the specific elements in the thin films, with the capability to probe charge transfer, orbital occupancy, magnetic excitations, and other electronic phenomena [250,251]. By selecting the X-ray energy, scattering geometry and wavevector transfer, RIXS can identify chemical states, bonding coordination, strain effects, and electronic structure variations across buried layers and interfaces with nanometer scale resolution. Time-resolved RIXS can also track ultrafast excited state dynamics. With tunable X-ray energies covering a wide range of absorption edges, RIXS reveals a wealth of electronic structure information complementary to photoemission and scattering techniques, making it invaluable for elucidating electronic properties and interactions governing thin-film and multilayer systems.

## 5. Summary and Future outlook

X-ray scattering and diffraction techniques provide critical insights into crystal structure, lattice spacing, epitaxial relations and texture in magnetic thin films. Small-angle X-ray scattering reveals magnetic domain sizes and correlations. Synchrotron-based X-ray spectroscopy elucidates element-specific magnetic properties with higher resolution. Advanced electron microscopy methods enable the direct imaging of magnetic domains, grains, defects and interfaces. Spectroscopy techniques help to analyze chemical composition and oxidation states at surfaces and interfaces. Neutron reflectometry provides depth-resolved magnetic structure profiling. Thus, by correlating macroscopic magnetic measurements with microscopic and spectroscopic characterization, fundamental structure–property relationships can be established in thin-film systems. Big data analytics and machine learning can accelerate knowledge discovery from multidimensional characterization datasets. Advanced simulations could guide interpretations. Overall, the continued development of operando techniques and multi-modal datasets would be helpful in providing deeper insight into magnetism in thin films.

## Figures and Tables

**Table 1:** General classification of thin magnetic films depending on their specific usage.

Classification	Examples	References
Perpendicular Magnetic Anisotropy Films	Pt/Co, Pd/Co, Ta/CoFeB, Fe/Pt,Fe/Pd, Co/Pt	[5,6,7]
Magnetic Tunnel Junction Films	CoFeB/MgO/CoFeB and CoFeB/AlOx/CoFeB, MnGa/Co_2_MnSi	[8,9,10]
Topological Insulator Thin Films	Cr doped (Bi,Sb)_2_Te_3_, (Zn,Cr)Te/BST/(Zn,Cr)Te, MnBi_2_Te_4_/Bi_2_Te_3_	[11,12,13,14,15,16]
Heusler Alloy Thin Films	Co_2_FeAl, Co_2_MnGa, NiMnSb, Co_2_(Cr,Fe)Al	[17,18,19]
Spin Hall Effect Materials	Pt, W, Mn_3_Sn, Cr	[20,21,22,23]
Dilute Magnetic Semiconductors	GaMnAs, InMnAs, Fe-doped SiGe, Ni-doped ZnO	[24,25,26,27]
Magnetic Oxide Thin Films	magnetite (Fe_3_O_4_), hematite (Fe_2_O_3_), and cobalt ferrite (CoFe_2_O_4_), La_0.7_Sr_0.3_MnO_3_/SrIrO_3_, Pt/Tm_3_Fe_5_O_12_	[28,29,30,31]
Rare Earth–Transition Metal Thin Films	GdCo, TbFe, HoCo, TbDyFe, SmFeB	[22,32,33,34,35]
Antiferromagnetic Thin Films	Mn_2_Au and Mn_3_X (X = Ge, Sn)	[36,37,38]
Magnetic Multilayers	exchange-coupled layers, patterned magnetic nanostructures and artificial multiferroic structures	[39,40,41,42,43]

**Table 2:** Summary of various techniques for structural and morphological characterization of thin films.

Class	Technique	Probe	Information	Facility	References
Imaging	TEM, STEM, XTEM	Transmitted electrons	Provides morphological, structural, compositional and electronic information with atomic-scale resolution. Identifies crystal structures and orientations, visualizes defects, probes elemental maps and may visualize magnetic domains. TEM: A broad parallel beam illuminates the whole sample. STEM: Focused electron beams are rastered across the sample, and spatial variations in transmission, scattering or fluorescence are measured. XTEM: Thin cross-sections of the samples allow for the direct imaging of the atomic structure and chemistry at internal interfaces.	Lab.	[59,61]
	SEM	Secondary electrons	Provides nanometer-scale imaging of morphology and crystallographic analysis of sample surfaces. Coupled with spectroscopic probes (i.e., EDS or EELS) enables elemental mapping. Spin-polarized SEM probes magnetic states.	Lab.	[63,64,65]
	ToF-SIMS	Secondary ions	The obtained mass spectra and images can confirm the surface homogeneity in the thin films. Depth profiling through sputtering enables 3-dimensional representation of cation distribution at nanometer resolution.	Lab.	[66,67,68,69]
	SPLEEM	Backscattered electrons	Uses spin-polarized low-energy electrons to image magnetic domain structures and spin orientations on sample surfaces.	Sync.	[70,71,72]
	PEEM	Electrons	Exploits X-ray-excited photoelectrons to image magnetic domains and spin dynamics with high spatial and temporal resolution.	Sync.	[73,74]
	AFM/MFM	Current/Magnetostatic forces	Measures short-range forces between the tip and sample surface, enabling real-space imaging and the analysis of topography, mechanical, electrical and magnetic properties at nanometer to sub-nanometer resolution.	Lab.	[75,76,77]
Diffraction & Scattering	XRR	Reflected X-rays	Measures the reflection of X-rays at shallow angles to probe layer thickness, roughness, density and buried interfaces with sub-nanometer precision.	Lab. Sync.	[78,79,80,81,82]
	A-XRR	Reflected X-rays	Adds chemical selective information, exploiting the anomalous scattering effect. Effective when the X-ray energy is tuned close to an absorption edge in the sample. Exploiting the XMCD effect allows for the provision of element-specific magnetic information.	Sync.	[83,84,85,86,87,88]
	XRD, HRXRD	Diffracted X-rays	Provides crystallographic information about the crystallographic structure, strain, disorder and coherence length of crystallites. It is possible to exploit the anomalous scattering effect to obtain element-specific structural information.	Lab. Sync.	[89,90,91]
	GIXRD, GISAXS	Diffracted X-rays	By analyzing diffuse X-ray scattering, structural information can be obtained for buried layers and interfaces.	Lab. Sync.	[92,93,94,95,96]
	ND	Diffracted neutron	Like XRD, ND provides crystallographic information about the crystallographic structure, strain, disorder and coherence length of crystallites. It is helpful especially for detecting lighter elements. Neutron sensitivity to the magnetic moment allows for the direct probing of the magnetic structure of buried layers and interfaces. Available only at synchrotron radiation facilities.	Sync.	[97,98,99]
	NR	Reflected neutron	Like XRR, NR probes layer thickness, roughness, density and buried interfaces with sub-nanometer precision. It can exploit neutron sensitivity to obtain information about magnetic structures. Available only at synchrotron radiation facilities.	Sync.	[97,99,100,101]
	PNR	Reflected neutron	The spin dependence of neutron scattering can reveal the buried magnetic layer structure and interfacial magnetic profiles. Available only at synchrotron radiation facilities.	Sync.	[102,103,104]
	CDI	Scattered coherent X-rays	Measures the diffraction pattern produced when a coherent beam of X-rays (or electrons) interacts with a sample to visualize the internal structure of objects at the nanoscale. Strain mapping is possible.	Sync.	[105,106,107]
	XSW, RXSW	Interference between incident and reflected X-rays	When an X-ray beam is reflected at a shallow angle, it forms a standing wave pattern. By tuning to resonant modes, the XSW field probes precise depths. Combined with XRF, XRD, XAFS and XMCD, it adds depth-selective information to these techniques. Tuning the X-ray energy to an absorption edge enhances the standing wave field at the resonant atomic species, providing elemental and depth specificity. Some key advantages of XSW combined with XRF, XRD, XAFS, and XMCD are the possibility to probe light elements, highly diluted elements, and a depth resolution down to the nanometer or sub-nanometer scale, making them ideal complements to neutron- and electron-based techniques for studying buried layers and interfaces in thin-film magnetic systems. Proper modeling is needed to translate the measured signals into quantitative depth profiles.	Lab. Sync.	[79,108,109,110]
Spectroscopies	XAFS, P-XAFS	Absorbed X-ray photons	Provides chemically selective information about local atomic structure, valence states and magnetic properties through dichroism effects. Available only at synchrotron radiation facilities. Polarized XAFS adds some directional sensitivity to the XAFS techniques.	Sync.	[111,112]
	XMCD	Absorbed X-ray photons	Element-specific magnetic properties and detailed spin-ordering information. Available only at synchrotron radiation facilities.	Sync.	[113,114]
	PES, XPS	Photoelectrons	Measures electronic band structure and chemical composition, and probes occupied electronic states and chemical composition at surfaces and interfaces. Ambient pressure XPS enables in situ studies.	Lab. Sync.	[115,116,117,118]
	Spin-polarized PES	Photoelectrons	Probes the energy and spin polarization of the photoemitted electrons, providing insights into the electronic structure and magnetic properties.	Sync.	[74]
	ARXPS	Photoelectrons	Measures the energy and emission angle of photoemitted electrons to obtain information about their energy and momentum distribution. This provides valuable insights into a material’s band structure. Tuning the emission angle provides depth-sensitive chemical information.	Lab. Sync.	[119,120]
	XRF	Fluorescence X-rays	By tuning the X-ray energy to the absorption edges of different elements, XRF can provide elemental composition and chemical information from buried layers and interfaces. The fluorescence yield is measured as a function of the incident angle.	Lab. Sync.	[121,122,123,124]
	RIXS	Photons	Probes the re-emitted X-ray from specific energy levels. Provides chemical selective insights into electronic excitations, spin states and magnetism.	Sync.	[125,126,127]
	SXES	Photoelectrons	Consists of focusing an X-ray beam onto a sample and measuring the X-ray emissions. Probes the electronic structure and bonding characteristics from shallow depths.	Sync.	[128,129]

**Table 3:** AFM modes for functional properties with the physical quantity measured, and examples of information that can be acquired (adapted from https://www.spectraresearch.com/wp-content/uploads/2016/08/Thin-Films-Characterization-AFM.pdf (accessed on 18 November 2023)).

Mode	Sensing	Information Obtained	References
Conductive AFM	Current	conductivity, film uniformity and defects, dielectric breakdown, dopant distribution	[161]
Electrostatic Force Microscopy	Electrostatic Forces	electrostatic gradients, capacitance variations, embedded conductors	[161]
Kelvin Probe Force Microscopy	Electric Potential	surface potential, work function, film uniformity and coverage	[161]
Scanning Microwave Impedance Microscopy	Capacitance and Resistance	film thickness, dielectric constant, permittivity or conductivity variations, buried charge	[162]
Piezoresponse Force Microscopy	Electromechanical Response	piezoelectric domains, polarization vector and switching, ferroelectric coercive field	[163]
Magnetic Force Microscopy	Magnetostatic Forces	magnetic domains, magnetization hysteresis, magnetic coercive field	[76,77]

**Table 4:** Survey of different imaging techniques of magnetic domains.

Technique	Mechanism	Lateral Resolution (nm)	Depth Resolution (Å)	Element Selectivity	Reference
Transmission Electron Microscopy (TEM)	Lorentz Force Deflection	≥50	-	No	[164]
Magneto-Optical Kerr Effect (MOKE)	Magneto-optical Effects	~500	≈200	No	[165]
Magnetic Force Microscopy (MFM)	Magnetic Dipole interaction	≥100	-	No	[166]
Scanning Near-field Optical Microscopy (SNOM)	Magneto-optical Effects	≥250	~100	No	[167]
Spin-polarized Scanning Tunnelling Microscopy (SP-STEM)	Spin-dependent Tunneling	≤1	~1	No	[168]
Spin-polarized LEEM	Exchange Scattering	≥50	~5	No	[139]
X-ray Transmission Microscopy (XTM)	Magnetic X-ray Dichroism	~50	-	Yes	[169]
X-ray Photoemission Electron Microscopy (XPEEM)	Magnetic X-ray Dichroism	~50	~20	Yes	[170]
Ultra-violet Photoemission Electron Microscopy (UVPEEM)	Magnetic Linear Dichroism	~50	~20	No	[171]

**Table 5:** Basic difference in the characterization of thin films using GIXRD and GISAXS.

	GIXRD	GISAXS
Information Obtained	Study of the crystal structure and orientation of thin films. Determines the lattice parameters, crystal phases, crystallinity and crystallographic orientation in the film.	Investigates the nanostructures and surface morphology of thin films. Information about the size, shape, spatial arrangement and interactions of nanoscale features on the film surface. Also detects the presence of nanoparticles, pores, phase separation, self-assembly and other nanoscale phenomena.
Measurement Principle	Involves measuring the scattering of X-rays by the crystal planes in the thin film.	The incident X-rays interact with the nanostructures on the thin film surface, resulting in scattering in different directions.
Suited For	Suitable for studying epitaxial layers, polycrystalline films and crystalline structures in general.	Suitable for studying thin films with nanoscale features, including ultrathin films and films having a non-crystalline or amorphous structure.

**Table 6:** Representation of the information that can be obtained for different types of thin films from X-ray diffraction.

Structure of Thin Film	Thickness	Composition	Lattice Strain	Residual Stress	Defects	Crystal Orientation	Surface Roughness	Lateral Structure
Perfect Epitaxial	HRXRD	HRXRD	100%	-	-	HRXRD	XRR	XRR
	XRR							
Textured Polycrystalline	XRR	XRPD	GIXRD	GIXRD	XRPD	GIXRD	XRR	XRR
		GIXRD			GIXRD	GISAXS		GISAXS
Polycrystalline	XRR	XRPD	-	GIXRD	XRPD	GISAXS	XRR	XRR
		GIXRD			GIXRD			GISAXS

## List of Abbreviations

AES	Auger Electron Spectroscopy
APECS	Auger-photoelectron coincidence spectroscopy
AFM	Atomic Force Microscopy
ALD	Atomic Layer Deposition
ARXPS	Angle-resolved X-ray Photoelectron Spectroscopy
A-XRD	Anomalous (Resonant) X-ray Diffraction
A-XRR	Anomalous (Resonant) X-ray Reflectivity
BSE	Backscattered Electron
CDI	Coherent Diffractive Imaging
CBED	Convergent-beam Electron Diffraction
CTR	Crystal Truncation Rod
CVD	Chemical Vapor Deposition
EBSD	Electron Backscatter Diffraction
EDS	Energy-dispersed Spectroscopy
EELS	Electron Energy Loss Spectroscopy
EXAFS	Extended X-ray Absorption Fine Structures
FIB	Focused Ion Beam
GIXRD	Grazing Incidence X-ray Diffraction
GISAXS	Grazing Incidence Small-angle Scattering
GIXRF	Grazing incidence X-ray Fluorescence
HAADF	High-angle Annular Dark Field
HRCTRS	High-resolution Crystal Truncation Rod Scattering
HRTEM	High-resolution Transmission Electron Microscopy
HRXRD	High-resolution X-ray diffraction
LEED	Low-energy Electron Diffraction
MExFM	Magnetic Exchange Force Microscopy
MFM	Magnetic Force Microscopy
MBE	Molecular Beam Epitaxy
ND	Neutron Diffraction
NR	Neutron Reflectivity
PDF	Pair Correlation Functions
PEEM	Photoemission Electron Microscopy
PES	Photoelectron Spectroscopy
PLD	Pulsed Laser Deposition
PMA	Perpendicular Magnetic Anisotropy
RHEED	Reflection High-energy Electron Diffraction
RSM	Reciprocal Space Map
RIXS	Resonant Inelastic X-ray Scattering
R-XSW	Resonant X-ray Standing Wave
R-XRD	Resonant X-ray Diffraction
R-XRR	Resonant X-ray Reflectivity
SEM	Scanning Electron Microscopy
SXES	Soft X-ray Emission Spectroscopy
SPLEEM	Spin-polarized Low-energy Electron Microscopy
SP-PES	Spin-polarized Photoemission Spectroscopy
SP-STM	Spin-polarized Scanning Tunneling Microscopy
STEM	Scanning Transmission Electron Microscopy
TEM	Transmission Electron Microscopy
ToF-SIMS	Time-of-flight Secondary Ion Mass Spectrometry
XAFS	X-ray Absorption Fine Structure
XAS	X-ray Absorption Spectroscopy
XANES	X-ray Absorption Near Edge Structure
XES	X-ray Emission Spectroscopy
XMCD	X-ray Magnetic Circular Dichroism
XPS	X-ray Photoemission Spectroscopy
XRD	X-ray Diffraction
XRF	X-ray Fluorescence
XRR	X-ray Reflectivity
XRS	X-ray Scattering
XSW	X-ray Standing Waves
SW-XAFS	Standing Waves–X-ray Absorption Fine Structure
XTEM	Cross-sectional Transmission Electron Microscopy
